# Atmospheric Cold Plasma Induced Modification of the Carob Germ Protein Isolate

**DOI:** 10.3390/foods15213896

**Published:** 2026-10-30

**Authors:** Halil İbrahim Odabaş

**Affiliations:** Department of Food Engineering, Faculty of Agriculture, Eskişehir Osmangazi University, 26160 Eskişehir, Türkiye; odabasibrahim@gmail.com

**Keywords:** carob germ, protein, techno-functionality, structure

## Abstract

Carob germ is a protein-rich industrial by-product derived from locust bean gum processing. This study evaluated the potential of atmospheric cold plasma (CP) as a physical modification strategy to improve the structural and techno-functional properties of the protein isolated from carob germ (CGP) over treatment durations of 5, 10, and 15 min. SDS-PAGE and FTIR analyses revealed that CP treatment preserved the subunit composition and main functional groups of CGP. However, deconvolution of the Amide I region demonstrated a shift from ordered β-sheets toward flexible random coils. 5-min CP exposure induced partial protein unfolding, which significantly increased surface hydrophobicity, free sulfhydryl content, and solubility. Simultaneous TGA/DSC analysis indicated that short-term CP treatment altered the thermal response of CGP, with the 5-min treatment showing the highest thermal decomposition rate, whereas the highest denaturation temperature and enthalpy were observed after 10 min. Techno-functional evaluations revealed that 5-min CP exposure produced the highest emulsifying activity index and a 2.1-fold increase in emulsion stability index. Extending CP treatment to 10 min maximized the foaming capacity, whereas prolonged 15-min exposure led to reduced solubility and emulsifying performance while maximizing water holding capacity. Overall, moderate CP treatment (5 min) serves as an effective, eco-friendly physical modification method to enhance the techno-functionality of CGP.

## 1. Introduction

Carob (*Ceratonia siliqua*) is extensively used in the food, cosmetic, and pharmaceutical industries primarily due to the high galactomannan content extracted from its seeds [1]. Carob galactomannan, also known as locust bean gum, is a creamy white powder obtained by milling the endosperm of the carob seed, which consists of 42–46% endosperm, 30–33% husk and 23–25% germ, by seed weight [2]. Although the endosperm of the carob seed is the primary source for commercial gum, the germ fraction remains largely underutilized despite its high nutritional value, as it contains protein concentrations that can exceed 50% [3]. The high protein content of carob germ underscores its promise as a sustainable and high-value plant-based protein source.

Plant-based proteins have gained increasing interest in recent years due to their relatively low environmental impact compared to animal-based proteins and growing consumer demands in plant-based food ingredients. In current industrial practice, plant protein ingredients are utilized for specific functional roles in product formulations such as water- and oil-binding in meat gels and doughs, emulsification in plant-based creamers, and foaming in whipped toppings. However, the incorporation of the plant-proteins into the food systems are often limited by their low techno-functional properties such as solubility, foaming, emulsifying and gelling [4]. To address these drawbacks, a wide range of modification methods has been explored to tailor and optimize the techno-functional performance of plant-derived proteins. These modification methods can be classified into chemical, enzymatic and physical methods. Despite their effectiveness chemical and enzymatic methods may increase the complexity and cost of the process [5]. Physical modification methods such as ultrasonication [6], high-pressure homogenization [7], pulsed electric fields [8] and irradiation [9] have gained increasing attention as alternative approaches for improving the techno-functional properties of plant proteins.

In this regard, cold plasma (CP) processing has gained significant attention as a novel non-thermal physical modification tool to enhance the functional performance of proteins. CP is technology generated by applying electrical energy to a gas at atmospheric pressure, producing a partially ionized medium containing reactive oxygen and nitrogen species, electrons, ions, and excited species [10]. These highly reactive components can interact with protein molecules and induce structural changes through oxidation, unfolding, aggregation [11]. Such modifications can influence protein conformation, surface properties, and intermolecular interactions, thereby affecting important techno-functional properties such as solubility, emulsifying activity, foaming capacity, water-holding capacity, and gelation properties [12,13,14,15,16].

Despite the increasing interest in the application of physical modification methods to improve the techno-functional properties, such as aqueous solubility, emulsifying properties, foaming behavior and water/oil holding capacities of plant proteins, studies on the physical modification of carob germ protein isolate (CGP) remain limited. To the best of our knowledge, the effect of CP treatment on the structural and techno-functional properties of carob germ protein isolate has not yet been investigated. Therefore, the present study represents a novel approach to the physical modification of CGP using CP. This study aims to elucidate the effects of plasma treatment on the structural and techno-functional properties of CGP isolate and to assess its potential as a functional plant protein ingredient for food applications.

## 2. Materials and Methods

### 2.1. Materials

Carob germ sample was kindly provided by commercial locust bean gum manufacturer located at Mersin, Turkiye. All chemicals and reagents were of analytical grade and purchased from Sigma-Aldrich (St. Louis, MO, USA).

### 2.2. Protein Isolation from Carob Germ

Carob germ samples were ground into a fine powder using a laboratory mill. To remove lipids, the powder was defatted with n-hexane at a solid to solvent ratio of 1:10 (*w*/*v*). Defatting process was repeated twice. Following filtration, the residual solvent was evaporated under a fume hood for 24 h. CGP was isolated from defatted carob germ flour using the protocol described by Bengoechea, et al. [17] with some minor modifications. Defatted flour was dispersed in distilled water at a ratio of 1:15 (*w*/*v*), and the pH of the suspension was adjusted to 10.5 using 5 M NaOH. The mixture was stirred continuously with an overhead stirrer for 1 h, followed by centrifugation at 10.000× *g* for 15 min to separate the insoluble fraction. The resulting supernatant was collected, and pH was adjusted to 4 using 5 M HCl to isoelectric precipitation. The precipitated protein was washed twice with distilled water, recovered by centrifugation, re-suspended in distilled water and neutralized to pH 7.0 using 5 M NaOH. Finally, the neutralized protein suspension was freeze-dried using a freeze-dryer and stored for further treatments and analysis. Protein and moisture contents of the resultant CGP were 85.09 and 5.40, respectively.

### 2.3. Cold Plasma Treatment

**Figure 1:**
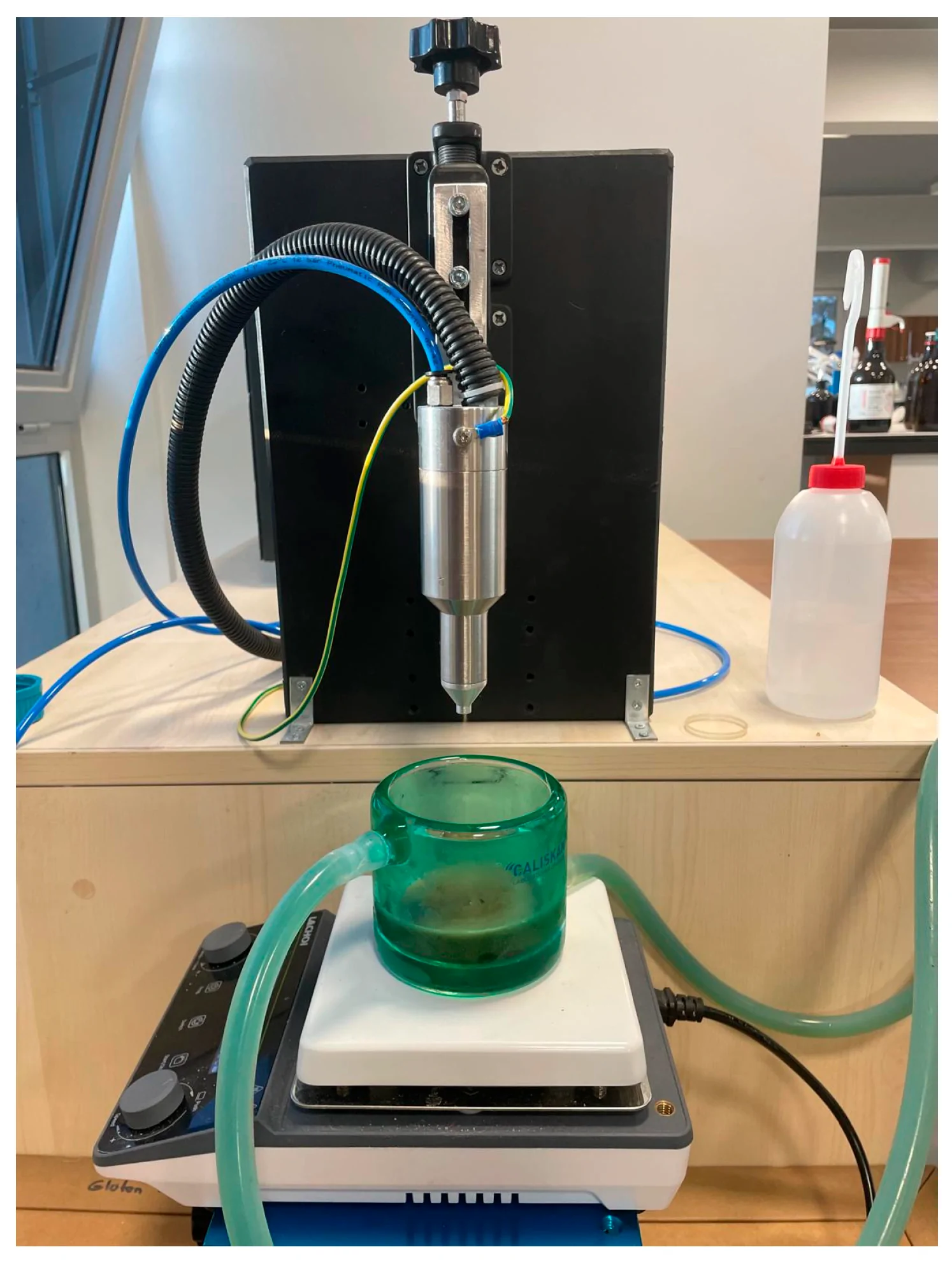
Atmospheric cold plasma treatment setup.

CP was applied to aqueous CGP dispersions. Protein dispersions were prepared by dissolving the protein in distilled water at a concentration of 10% (*w*/*v*) to a final volume of 50 mL. The dispersions were continuously stirred for 3 h to ensure complete hydration. Subsequently, the samples were transferred into a jacketed glass reactor connected to a circulating water bath maintained at 10 °C to minimize any temperature increase and prevent thermal damage during plasma treatment (Figure 1). To maintain homogeneous mixing throughout the process, the reactor (80 mm inner diameter) was placed on a magnetic stirrer set to 500 rpm, with the plasma probe positioned at a distance of 12 cm from the liquid surface. CP treatment was performed using an atmospheric pressure plasma jet system (300 W, 512–515 Hz, Plazmatek, Isparta, Türkiye), with ambient air used as the working gas at a constant flow rate of 10 L/min. The dispersions were exposed to plasma for 5, 10, and 15 min. Specific energy inputs (J/mL) were calculated as 1.8, 3.6 and 5.4 kJ/mL for the treatment durations of 5, 10 and 15 min, respectively. The surface temperature of the dispersion during CP treatment was monitored using an infrared thermometer (TFA Dotsman, Wertheim, Germany) and did not exceed 22 °C throughout the process. Following CP treatment, the samples were stirred for an additional 1 h to facilitate the dissolution of plasma-generated reactive species into the dispersions and to promote their interaction with the protein [18]. An untreated sample served as the control and was subjected to identical hydration, stirring, and post-treatment procedures, but without applying plasma discharge. Samples were then freeze-dried and stored in moisture tight bottles during the experiments.

### 2.4. Determination of the Structural Properties of the CGP Samples

#### 2.4.1. FTIR

FTIR spectra of the samples were recorded using an ATR-FTIR spectrometer (Alpha II, Bruker Optics, Ettlingen, Germany). The quantitative evaluation of protein secondary structure composition was performed by analyzing the amide I region (1600–1700 cm^−1^). A linear baseline correction was first applied between 1600–1700 cm^−1^ followed by spectral smoothing using a 7-point Savitzky-Golay filter. Deconvolution and curve-fitting of the Amide I band were performed using OriginLab software (version 2026b, OriginLab Corp., Northampton, MA, USA) with a Gaussian peak-fitting function. During the fitting procedure, the peak positions were held fixed at the specified frequencies, while the full width at half maximum of each peak was constrained between 5–35 cm^−1^. The component bands were assigned to secondary structure elements as follows: β-sheet (1620, 1632, and 1688 cm^−1^), random coil (1642 cm^−1^), α-helix (1654 cm^−1^), and β-turn (1670 cm^−1^). Iterative fitting was performed until a coefficient of determination (R^2^ > 0.999) was achieved. Relative contribution of each secondary structural component was calculated based on the corresponding integrated peak areas.

#### 2.4.2. Surface Hydrophobicity

Surface hydrophobicities (H_0_) of the CGP samples was determined using 1-anilino-8-naphthalenesulfonate (ANS) according to the method described by Zhu, et al. [19] with minor modifications. Protein stock solutions (10 mg/mL) were prepared in 0.01 M phosphate buffer (pH 8.0) and stirred at 300 rpm for 1 h at room temperature. Subsequently, a series of protein dilutions ranged from 0.01 to 0.05 mg/mL was prepared. For the fluorescence measurements, 20 µL of ANS solution (8.0 mM) was added to each protein solution, followed by incubation for 15 min. The fluorescence intensity was measured at an excitation wavelength of 390 nm and an emission wavelength of 470 nm using a slit width of 2.5 nm (RF-6000, Shimadzu, Japan). The H_0_ values were obtained from the initial slope of the fluorescence intensity and protein concentration.

#### 2.4.3. Free Sulfhydryl Groups

The content of free sulfhydryl (-SH) groups in native and CP treated CGP samples were determined according to the method of Ellman [20] with slight modifications. CGP samples were dispersed in phosphate buffer (0.1 M, pH 7.0) containing 1 mM EDTA and centrifuged at 8.000 rpm for 15 min. The soluble protein concentration in the supernatant adjusted to 0.1 mg/mL with the same buffer. Then, 3 mL of the CGP solution was mixed with 0.03 mL of Ellman’s reagent (10 mM DTNB in phosphate buffer), vortexed, and incubated for 15 min in the dark. The absorbance was recorded at 412 nm using a UV-Vis spectrophotometer (V-730, Jasco, Tokyo, Japan) against a reagent blank. Free -SH content was calculated using the molar extinction coefficient of 13.600 M^−1^cm^−1^ and expressed as μmol/g of CGP.

#### 2.4.4. SDS-PAGE

Native and CP treated CGP samples were analyzed by sodium dodecyl sulfate-polyacrylamide gel electrophoresis (SDS-PAGE) [21]. Briefly, CGP was mixed with sample buffer containing SDS, glycerol, 0.5 M Tris-HCl (pH 6.8), bromophenol blue, and 2-mercaptoethanol, followed by heating at 95 °C for 5 min to denature the protein. Equal amounts of protein samples and a molecular weight marker (10–250 kDa) were loaded onto a polyacrylamide gel (4% stacking, 12% resolving) and electrophoresed in running buffer at a constant voltage of 100 V until separation was achieved. Following electrophoresis, the gels were stained with Coomassie Brilliant Blue solution (0.1% CBB, 40% methanol, 7% acetic acid) to visualize the separated protein bands and subsequently destained with a methanol/acetic acid solution (40:7, *v*/*v*) until a clear background was obtained.

#### 2.4.5. Thermal Analysis

Thermal characterization of native and CP-treated CGP was performed using a simultaneous TGA/DSC analyzer (STA 200 Nexta, Hitachi, Japan). Samples (6–8 mg) were placed in perforated alumina pans and heated from 30 to 500 °C at 10 °C/min under a nitrogen purge, against an empty reference pan. Mass loss and heat flow curves were acquired simultaneously.

### 2.5. Determination of the Techno-Functional Properties of the CGP Samples

#### 2.5.1. Protein Solubility

CGP dispersions (%1, *w*/*v*) in phosphate buffer (pH 7.0, 0.1 M) were stirred at room temperature for 1 h using a magnetic stirrer and centrifuged at 10.000× *g* for 30 min at 20 °C. Protein content of the resultant supernatant was measured using the Bradford method [22]. Solubility was expressed as g protein/100 g CGP.

#### 2.5.2. Water/Oil Holding Capacity

The water holding capacity (WHC) and oil holding capacity (OHC) of the samples were determined following a modified centrifugal method [23]. Briefly, 0.1 g of CGP sample (m_0_) was weighed into a pre-weighed centrifuge tube (m_1_) and mixed with 2 mL of distilled water (for WHC) or sunflower oil (for OHC). The dispersion was thoroughly vortexed for 1 min to ensure complete wetting of the matrix and subsequently incubated for 30 min. Following incubation, the mixtures were centrifuged at 3.000× *g* for 20 min to facilitate phase separation. The supernatant was decanted, and the tubes were drained onto filter paper for 15 min to drain the remaining unabsorbed fluid. The tubes containing the wet pellet were then re-weighed (m_2_). The WHC and OHC values were expressed as grams of water or oil retained per gram of dry sample (g/g) using the following equation:

$$\mathrm{WHC}/\mathrm{OHC}\left(g/g\right)=\frac{m_{2}-m_{1}-m_{0}}{m_{0}}$$

#### 2.5.3. Emulsification Properties

Emulsifying properties were evaluated following the method described by Zhang, et al. [24], with minor modifications. A mixture containing 10 mL of sunflower oil and 30 mL of a 1% (*w*/*v*) protein solution was prepared and homogenized using a high shear homogenizer (T18, IKA, Germany) at 10.000 rpm for 2 min. Immediately after emulsification (0 min) and 10 min after emulsification, 50 μL aliquots were collected from the bottom of the emulsion and diluted with 5 mL of 0.1% SDS solution. The absorbance of each diluted sample was measured at 500 nm using a UV-Vis spectrophotometer (V-730, Jasco, Japan). The absorbance values obtained immediately after homogenization (A_0_) and after 10 min (A_10_) were used to determine the emulsifying activity index (EAI) and emulsion stability index (ESI), respectively.

EAI(m2g)=2×2.303×A0×DF 0.25×PC×10,000

$$\mathrm{ESI}\;\left(\min\right)=\frac{A_{0\;}\times ∆t}{∆A}$$

where, A_0_ represents the initial absorbance of the emulsion measured at 500 nm; DF is the dilution factor; PC denotes the CGP concentration as g/mL; Δt represents the time interval between the initial and final measurements; and ΔA is the difference in absorbance between the initial and final measurements.

#### 2.5.4. Foaming Properties

Foaming properties (foaming capacity and foam stability) of the CGP samples were measured using the method described by Acharjee, et al. [25] with some minor modifications. Briefly, 0.3 g CGP sample was dispersed in 30 mL of distilled water within a 100 mL graduated cylinder. The dispersion was homogenized using a high-shear homogenizer (T18, IKA, Staufen, Germany) at 10.000 rpm for 2 min. The initial (V_0_) and final (V_f_) volumes of the dispersion were recorded to calculate the foaming capacity (FC) using following equation:

$$\mathrm{FC}\;\left(\%\right)=\;\frac{V_{f}-V_{0}}{V_{0}}\;\times \;100$$

To evaluate the foam stability (FS), graduated cylinder was allowed to stand undisturbed for 30 min, remaining volume (V_30_) was measured. FS was calculated using the following equation:

$$\mathrm{FS}\;\left(\%\right)=\;\frac{V_{30}-V_{0}}{V_{f}-V_{0}}\;\times \;100$$

### 2.6. Statistical Analysis

All experiments were performed in analytical triplicates (n = 3) and the results were expressed as mean ± standard deviation. One-way analysis of variance (ANOVA) followed by Duncan’s multiple range test (*p* < 0.05) was applied to determine significant differences among groups using SPSS software (version 26.0).

## 3. Results and Discussion

### 3.1. Effects of Cold Plasma Treatment on the Structural Properties of CGP

**Figure 2:**
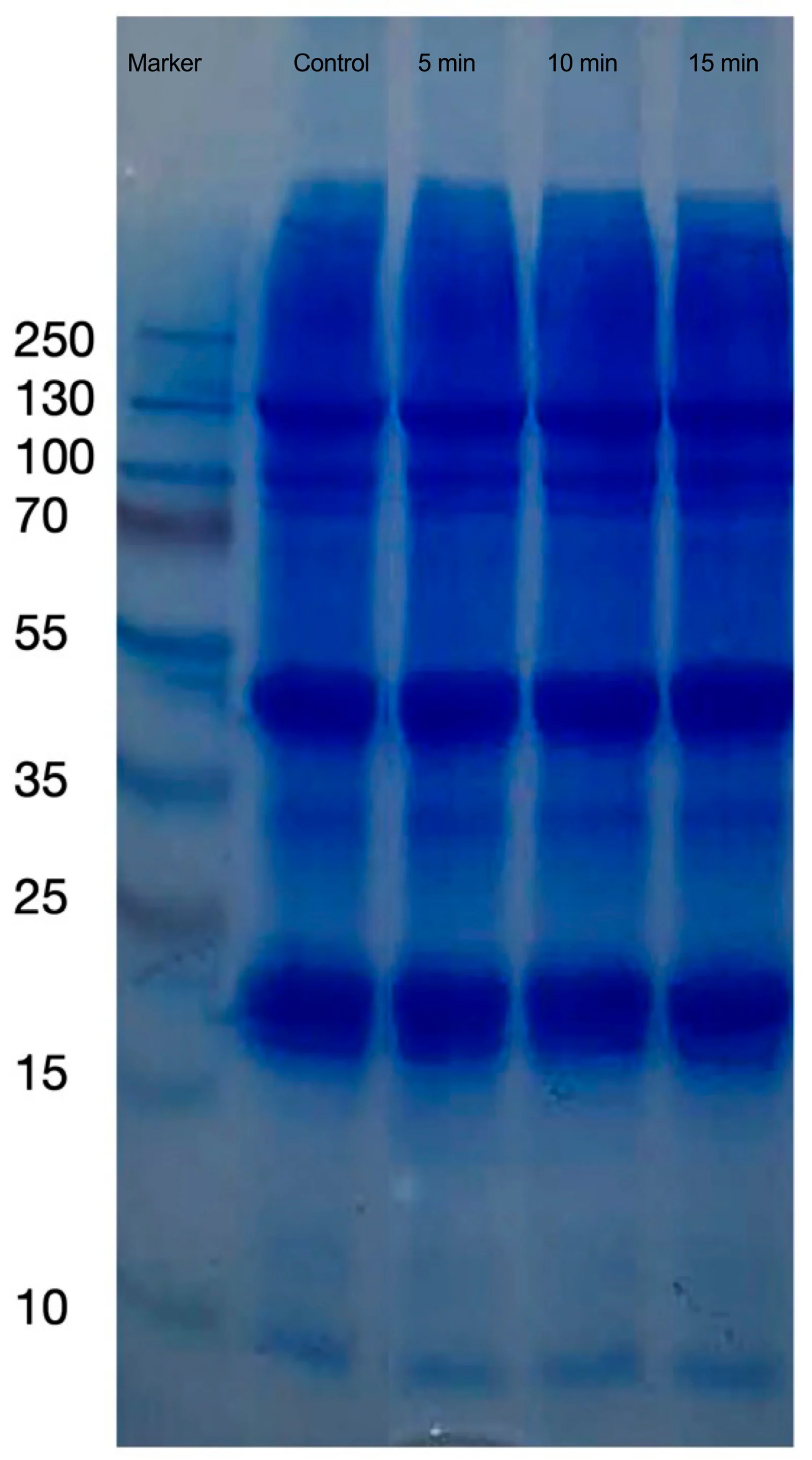
SDS-PAGE analysis of CGP samples.

The effects of CP treatment on the protein profiles of the CGP samples were evaluated using SDS-PAGE. As shown in the Figure 2, the control sample exhibited several distinct protein bands particularly at 130, 100, 35–55 and 15–25 kDa. The observed electrophoretic profile is consistent with previously reported SDS-PAGE patterns of carob germ proteins. Bengoechea, Romero, Villanueva, Moreno, Alaiz, Millán, Guerrero and Puppo [17] reported four major protein fractions at approximately 131, 102, 48, and 24 kDa under reducing conditions. Major bands observed in the control sample were also present in the CP treated CGP samples, indicating that the overall protein profile was largely preserved following the CP treatment. This observation is consistent with a study on sunflower seed protein, in which different CP treatment conditions produced only minor visual differences in SDS-PAGE subunit bands, while the overall protein and subunit composition remained unchanged [26]. Similarly, Zhang, et al. [27] demonstrated that CP treatment did not alter the molecular weight of native subunits (such as β-conglycinin and glycinin) nor generate distinct degradation fragments, confirming the preservation of the primary subunit structure of soy protein.

**Figure 3:**
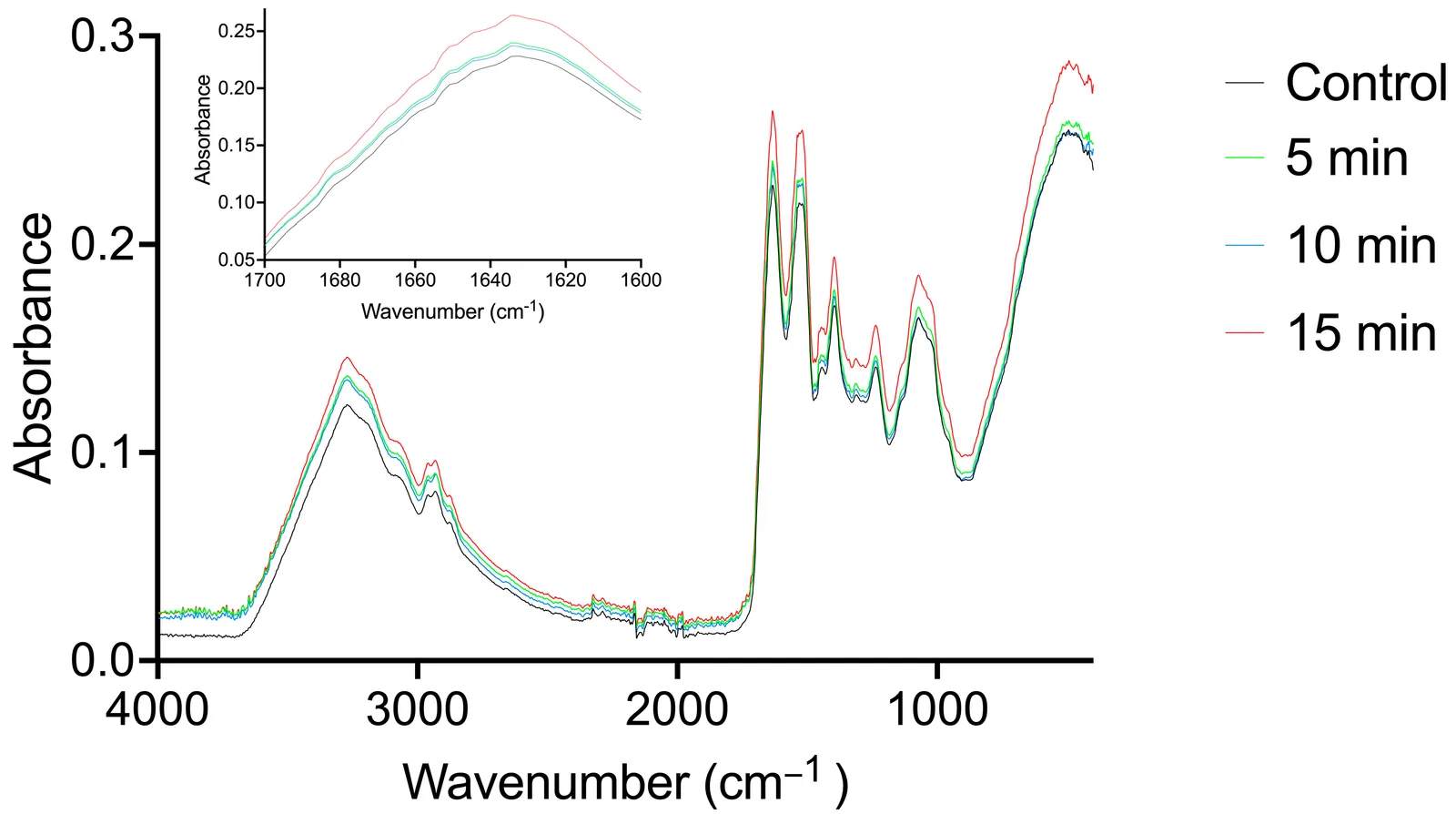
FT-IR spectrum of CGP samples.

FT-IR spectroscopy was used to examine the effects of CP treatment on the functional groups and secondary structure of carob germ proteins. The FTIR spectra and enlarged Amide I region (1700–1600 cm^−1^) of the CGP samples are shown in Figure 3. All samples displayed the typical absorption bands of proteins. The broad band near 3300 cm^−1^ was assigned to the Amide A region and was associated with N–H and O–H stretching vibrations. The bands at approximately 1636 and 1540 cm^−1^ corresponded to the Amide I and Amide II regions, respectively, while the bands between 1200 and 1400 cm^−1^ were related to Amide III vibrations [28]. CP treatment did not substantially change the positions of the Amide A, Amide I, or Amide II bands compared with the control. These results indicate that the main functional groups and chemical structure of the carob germ proteins remained intact after plasma treatment, consistent with the SDS–PAGE results.

**Figure 4:**
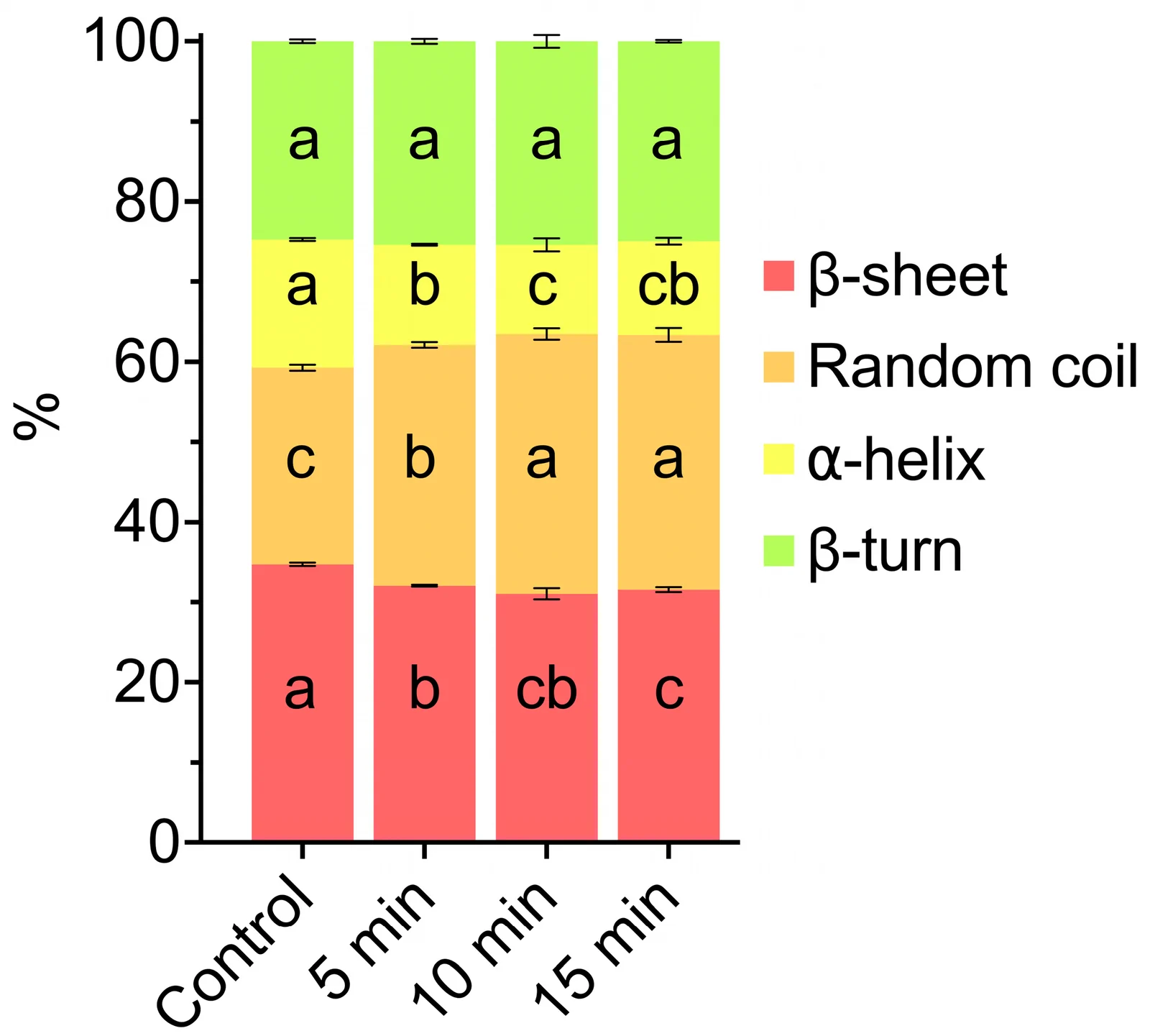
Secondary structure contents of CGP samples. (Different lowercase letters in the bars indicate statistically significant differences (*p* < 0.05)).

Figure 4 presents the quantitative distribution of secondary structure elements of CGP samples derived from the curve-fitting analysis of the Amide I band. The baseline conformation of the protein, characterized by a dominant β-sheet structural arrangement, is consistent with findings reported by Mamone, et al. [29]. However, it was observed that, depending on the duration of CP treatment, this ordered structure decreased in a statistically significant manner and gave way to disordered structural forms. Upon exposure to CP, a progressive structural transition is clearly observed. The percentage of the ordered β-sheet conformation showed a statistically significant decrease (*p* < 0.05) as the treatment duration increases. Concurrently, a significant increase is observed in the random coil fraction, while the α-helix content exhibits a moderate reduction and the β-turn structures remain relatively stable across the treatments. The findings clearly demonstrate that CP treatments disrupt ordered structures within the CGP matrix, transforming them into more flexible forms. This mechanism directly aligns with results reported by Rout and Srivastav [30] for soy and pea protein isolates, and by Zhang, et al. [31] for mulberry leaf proteins, regarding the decrease in α-helix and β-sheet contents and the consequent increase in disordered structures.

**Figure 5:**
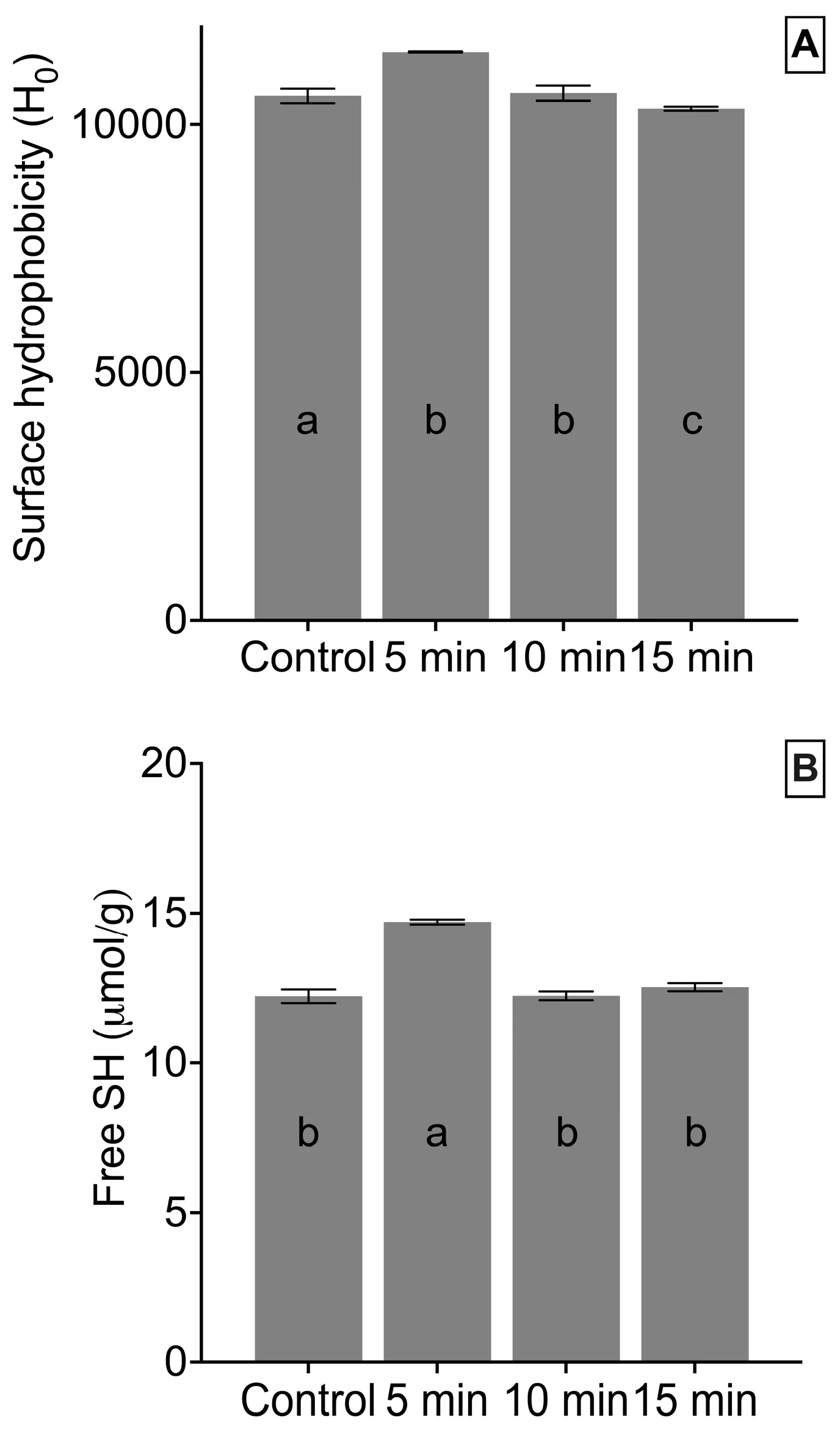
Surface hydrophobicity (**A**) and free -SH contents (**B**) of CGP samples. (Different lowercase letters in the bars indicate statistically significant differences (*p* < 0.05)).

Surface hydrophobicity (H_0_) results of the CGP samples are shown in the Figure 5A. A short period of CP treatment induced a significant increase (*p* < 0.05) in the H_0_, reaching the maximum value among all treatments including control. Increasing treatment duration to 10 min led to a slight decline in H_0_, which showed no statistically significant difference compared to the 5 min CP treated sample (*p* > 0.05). However, prolonged CP treatment for 15 min resulted in a significant reduction (*p* < 0.05) in the H_0_, which was significantly lower than both the CP treated samples (5 and 10 min) and control. The initial increase in H_0_ observed after 5 min of CP treatment can be attributed to the energetic bombardment of plasma-generated active species and high-energy particles.

This process is suggested to dissociate larger protein aggregates into smaller units and induce partial structural unfolding, thereby facilitating the exposure of buried hydrophobic residues toward the exterior surface [26]. Conversely, under extended CP exposure (e.g., 15 min treatment), the reduction in surface hydrophobicity may be associated with the clustering and non-covalent re-aggregation of protein molecules into larger macro-aggregates. In this context, previously exposed hydrophobic residues are hypothesized to become re-buried within the interior core of the newly rearranged aggregated structures, a plausible mechanism consistent with observations in similar plasma-treated protein systems [32,33].

The changes in the free sulfhydryl (-SH) content of CGP samples as a function of CP treatment time are presented in Figure 5B. Statistically significant differences (*p* < 0.05) were observed across the treatment durations. The control sample showed a baseline free -SH content of 12.23 μmol/g. Short-term CP exposure for 5 min induced a significant increase (*p* < 0.05) in the free -SH content, reaching the maximum value of 14.71 μmol/g. However, extending the CP treatment to 10 min and 15 min led to a decrease in free -SH levels to 12.24 μmol/g and 12.53 μmol/g, respectively. The free -SH values at 10 and 15 min were statistically non-significant from each other (*p* > 0.05), but were significantly lower (*p* < 0.05) than the 5 min CP treated CGP. The initial increase in free -SH content observed at 5 min of CP treatment is indicative of partial tertiary structure unfolding. The bombardment of energetic plasma species breaks non-covalent interactions, exposing internal, buried sulfur containing amino acid residues to the protein surface [34,35]. Conversely, extended CP application (10–15 min) resulted in a significant decline in free -SH content, dropping below the initial control levels. Prolonged exposure to atmospheric plasma generates high concentrations of reactive oxygen and nitrogen species, inducing an over-oxidative environment. Under these conditions, the exposed reactive free -SH groups may undergo oxidation, potentially contributing to the formation of covalent disulfide bonds or inter/intramolecular cross-links [33]. This oxidation-driven decrease in free -SH groups, along with increased hydrophobic exposure, is suggested to play a role in promoting protein re-aggregation [26,36].

**Figure 6:**
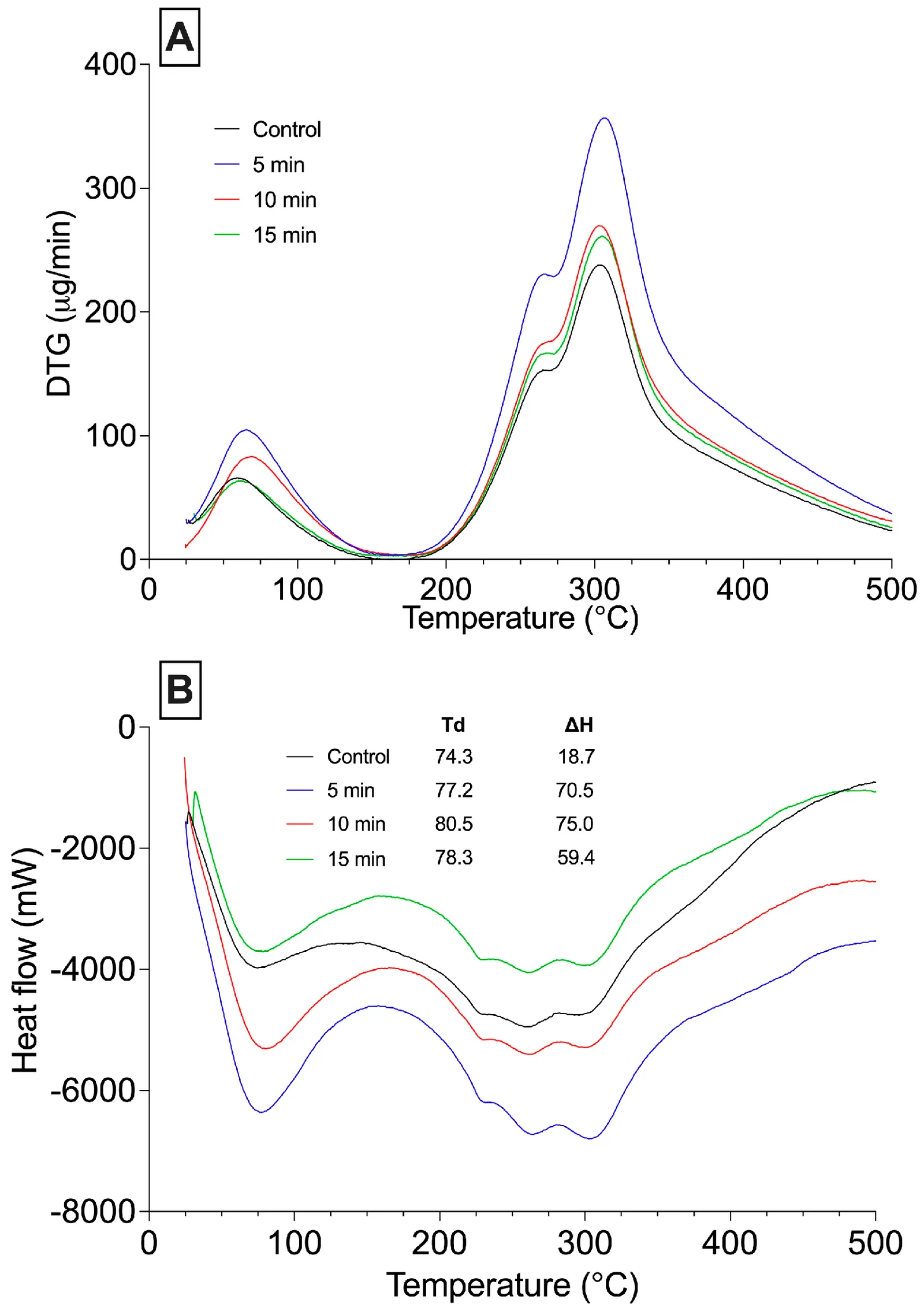
DTG (**A**) and DSC (**B**) curves of CGP samples.

The DTG and DSC curves (Figure 6A and Figure 6B, respectively) illustrate the thermal degradation behavior and thermal transition characteristics of control and CP treated CGP samples. The initial region (approximately 40–160 °C) was associated with overlapping dehydration and protein structural transitions. In particular, mass loss in this region can arise from the evaporation/desorption of water, while the accompanying DSC endothermic transition may reflect early-stage protein unfolding or denaturation. In this region mass loss rate in DTG increased from 65.7 μg/min in the control (T_max_ = 59.0 °C) to a peak value of 104.6 μg/min in the 5-min CP treated sample (T_max_ = 65.1 °C), before gradually declining to 82.8 μg/min at 10 min and 63.6 μg/min at 15 min. The DSC endothermic transition centered at 74.3–80.5 °C was assigned to protein thermal denaturation, although a contribution from dehydration cannot be completely excluded. The DSC thermograms revealed that the denaturation temperature (T_d_) shifted upward from 74.3 °C (control) to 77.2 °C (5 min), 80.5 °C (10 min), and 78.3 °C (15 min). A similar increase in T_d_ following CP treatment has been reported for plant protein isolates, including pea and sesame protein isolates, where the enhanced thermal stability was associated with stronger intermolecular interactions and protein cross-linking [37,38]. The corresponding denaturation enthalpy (ΔH) exhibited a substantial increase from 18.7 mJ/mg in native CGP to 70.5 mJ/mg at 5 min and reached a maximum of 75.0 mJ/mg at 10 min, followed by a decrease to 59.4 mJ/mg at 15 min. The second stage (200–400 °C) represents the primary thermal decomposition region where pyrolytic cleavage of the protein polypeptide backbone takes place. The DTG profiles exhibited a two-step degradation transition: a shoulder degradation step (263–266 °C) followed by a primary decomposition peak (302–305 °C). At the main decomposition peak, the 5-min CP treatment triggered the highest mass loss rate (356.4 μg/min at 305.4 °C), representing a nearly 50% increase over the native control (237.9 μg/min at 303.8 °C). Extended exposure (10 and 15 min) attenuated these decomposition rates to 269.8 μg/min and 261.2 μg/min, respectively.

### 3.2. Effects of Cold Plasma Treatment on the Techno-Functional Properties of CGP

**Figure 7:**
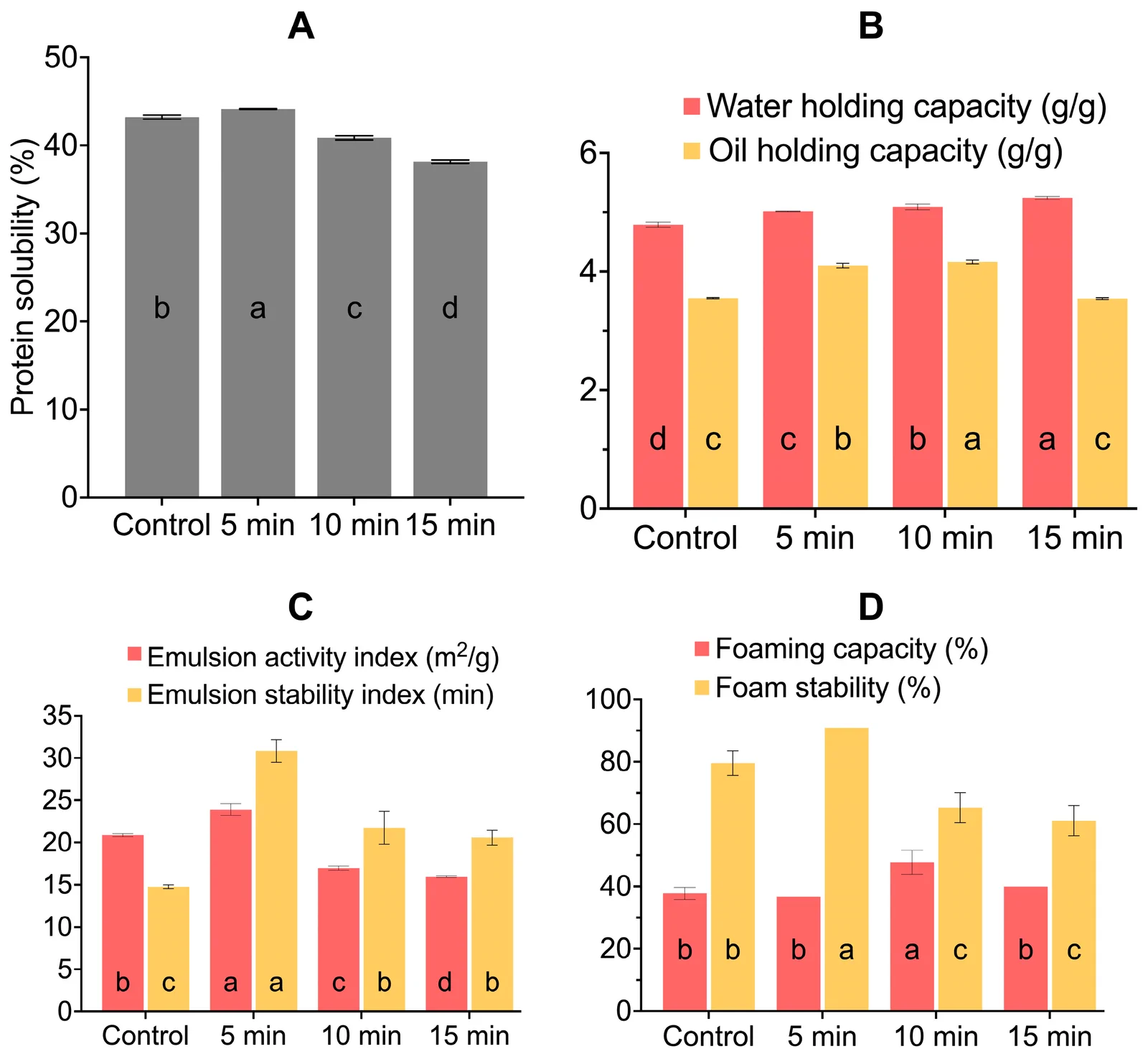
Techno-functional properties of CGP samples. (Different lowercase letters in the bars indicate statistically significant differences (*p* < 0.05)).

The effects of CP treatment on the techno-functional properties (protein solubility, water holding capacity, oil holding capacity, emulsion activity index, emulsion stability, foaming capacity and foaming stability) of CGP were shown in the Figure 7.

The effect of CP treatment on the protein solubility is presented in the Figure 7A. Statistically significant differences (*p* < 0.05) were observed among all groups. Control sample exhibited a baseline protein solubility of 43.22%. CP treatment of 5 min induced a significant increase in protein solubility, reaching the maximum value of 44.13%. However, the extended CP application to 10 and 15 min resulted in a progressive and significant decline in the protein solubility to 40.86% and 38.14%. The initial improvement in protein solubility after 5 min of CP treatment can be attributed to partial structural unfolding and the exposure of hydrophilic functional groups to the aqueous phase, driven by active species generated during plasma discharge. Conversely, the decline in solubility observed at 10 and 15 min CP treatments may be attributed to structural modifications induced by extended exposure to reactive species. Prolonged plasma exposure is hypothesized to promote partial conformational unfolding and the exposure of buried hydrophobic residues, which in turn facilitates non-covalent hydrophobic aggregation into larger, less soluble protein complexes [14,26,27].

The effects of CP treatment duration on the water holding capacity (WHC) and oil holding capacity (OHC) of the protein samples are presented in Figure 7B. Statistically significant differences (*p* < 0.05) were observed among the treatment groups for both parameters. The baseline WHC of the control sample was recorded as 4.79 g/g. CP treatment induced a significant increase (*p* < 0.05) in WHC with increasing exposure time. Specifically, WHC increased to 5.02 g/g at 5 min, 5.09 g/g at 10 min, and reached its maximum value of 5.24 g/g after 15 min of CP exposure. The progressive and statistically significant increase in the WHC of CGP with extended CP treatment duration can be directly explained to plasma-induced structural modification and molecular entrapment mechanisms. According to Rout and Srivastav [39] plasma treatment induces the unfolding of the native protein structure, which progressively expands the physical matrix of the protein and enhances its total capacity to bind water. This conformational opening is further driven by the generation of ionic and highly reactive species during CP discharge, which alter the local polar interactions and contribute to the augmentation of WHC [30]. Furthermore, Sharifian, et al. [40] reported that during CP treatment, the newly formed cross-links between protein molecules construct a three-dimensional network capable of physically trapping water molecules within its matrix. The OHC values exhibited an initial increasing trend followed by a decline at prolonged treatment times. The OHC of the control sample was 3.55 g/g. Exposure to CP for 5 min and 10 min significantly increased (*p* < 0.05) the OHC to 4.10 g/g and a maximum of 4.16 g/g, respectively. However, extending the CP treatment time to 15 min resulted in a sharp decrease in OHC (3.54 g/g), returning it to a level statistically non-significant from the control (*p* > 0.05).

The emulsification properties of CGP as a function of CP treatment duration was evaluated through the emulsion activity index (EAI) and emulsion stability index (ESI) (Figure 7C). CP processing significantly altered both parameters in a time-dependent manner (*p* < 0.05). Native CGP exhibited a baseline EAI of 20.90 m^2^/g. A 5 min CP treatment elevated the EAI to its peak value of 23.90 m^2^/g (*p* < 0.05). However, extending the plasma exposure beyond this threshold induced a progressive decline, with EAI dropping to 16.95 m^2^/g at 10 min and reaching its lowest level of 15.97 m^2^/g at 15 min, both falling below the native CGP. A more striking trend emerged in the ESI. The native protein displayed weak emulsion stability, with an ESI of only 14.77 min. Exposure to CP for 5 min resulted in a remarkable 2.1-fold enhancement in ESI, reaching a maximum of 30.83 min (*p* < 0.05). Although prolonged exposure (10–15 min) attenuated this stabilizing effect, reducing ESI to 21.73 min and 20.57 min respectively (*p* > 0.05 between 10 and 15 min), both values remained markedly superior to the untreated control (*p* < 0.05). The peak emulsifying performance observed at 5 min directly reflects the favorable structural changes identified in spectroscopic and surface analyses. Short term CP exposure delivers sufficient energy to break non-covalent constraints, driving the transition from rigid α-helices to flexible random coils while exposing previously buried hydrophobic core residues and free -SH groups. This heightened structural flexibility and amphiphilic surface character allow CGP molecules to rapidly migrate to, adsorb onto, and orientate across the oil-water interface during emulsification [41,42]. Conversely, the performance loss at 10- and 15-min CP treatments underscores the detrimental impact of plasma over exposure. Prolonged contact with reactive nitrogen and oxygen species promotes extensive protein unfolding and oxidation of functional groups, which may induce non-covalent re-aggregation as well as potential oxidative cross-linking [41,42,43]. These rigid, bulky complexes suffer from impaired diffusion kinetics and steric resistance, which hinders their ability to quickly align at the interface and lower interfacial tension hence the drop in EAI and ESI.

The influence of CP treatment on the foaming capacity (FC) and foam stability (FS) of CGP is illustrated in Figure 7D. Statistical evaluation revealed distinct, treatment time-dependent responses for both parameters (*p* < 0.05). The native CGP displayed a baseline FC of 37.78%. Short-term exposure to CP for 5 min did not induce a statistically significant change in FC (36.67%, *p* > 0.05). However, extending the treatment time to 10 min resulted in a sharp, statistically significant increase (*p* < 0.05), with FC reaching its maximum value of 47.78%. At 15 min of exposure, FC declined back to 40.00%, returning to a level statistically non-significant from the control group (*p* > 0.05). FS demonstrated an immediate positive response at shorter treatment durations. The native CGP exhibited an FS of 79.55%. A 5 min CP treatment elevated FS to its absolute peak of 90.91% (*p* < 0.05). Beyond 5 min, extended plasma application (10 and 15 min) led to a significant deterioration in FS, with dropping to 65.30% and 61.11%, respectively. Short term CP treatment induces controlled partial unfolding, exposing non-polar residues and -SH groups without causing severe aggregation. This optimized amphiphilic balance allows CGP to adsorb rapidly at the air-water interface, forming a flexible yet continuous viscoelastic film. This resilient protein network effectively retards liquid drainage and bubble coalescence, driving FS to its peak value [35]. However, as extended plasma exposure (10–15 min) continues to generate reactive oxygen species, potential oxidative cross-linking via disulfide bond formation becomes dominant. The resulting insoluble macro-aggregates, while still flexible enough at 10 min to encapsulate air, form a rigid and non-uniform interfacial film that lacks sufficient elasticity [31]. Consequently, the foam lamellae become brittle and susceptible to rapid liquid drainage, causing FS to drop significantly at 10 and 15 min. By 15 min, excessive oxidative re-aggregation severely restricts molecular mobility and protein solubility altogether, resulting in a concurrent decline in both foaming capacity and foam stability [31,43].

## 4. Conclusions

This study demonstrated that CP can effectively modify the structural and techno-functional properties of CGP. SDS-PAGE and FTIR analyses indicated that CP treatment largely preserved the primary protein profile and major functional groups, whereas it was observed that the disruption of ordered secondary structures, characterized mainly by a decrease in β-sheet content and an increase in random coil structures. These findings indicate that CP primarily induced conformational rearrangement rather than extensive degradation of the CGP polypeptide chains. Overall, the findings demonstrate that the functional properties of CGP can be modulated through controlled CP exposure, with 5 min identified as the most effective treatment for improving techno-functional performance, particularly emulsifying properties, while 10 min provided the greatest resistance to thermal denaturation. It should be noted that this optimal treatment time of 5 min is explicitly bounded by the exposure range tested. Therefore, CP represents a promising non-thermal and environmentally friendly approach for tailoring CGP for potential application as a functional plant-protein ingredient. Nevertheless, optimization of plasma parameters beyond treatment time, together with direct characterization of oxidative modifications, would be valuable for establishing more precise structure-function relationships and defining the optimal processing conditions for specific food applications.

## Data Availability

The original contributions presented in this study are included in the article. Further inquiries can be directed to the corresponding author.
